# Advanced research on vasculogenic mimicry in cancer

**DOI:** 10.1111/jcmm.12496

**Published:** 2015-01-19

**Authors:** Lili Qiao, Ning Liang, Jiandong Zhang, Jian Xie, Fengjun Liu, Deguo Xu, Xinshuang Yu, Yuan Tian

**Affiliations:** a Department of Oncology, Shandong University School of MedicineJinan, Shandong Pro, China; b Department of Radiation Oncology, Qianfoshan Hospital Affiliated to Shandong UniversityJinan, Shandong Pro, China; c Department of Oncology, Zhangqiu People's HospitalJinan, Shandong Pro, China

**Keywords:** vasculogenic mimicry, molecular regulation, cancer stem cell, tumour-target therapy, prognosis, high aggressive tumour

## Abstract

Vasculogenic mimicry (VM) is a brand-new tumour vascular paradigm independent of angiogenesis that describes the specific capacity of aggressive cancer cells to form vessel-like networks that provide adequate blood supply for tumour growth. A variety of molecule mechanisms and signal pathways participate in VM induction. Additionally, cancer stem cell and epithelial-mesenchymal transitions are also shown to be implicated in VM formation. As a unique perfusion way, VM is associated with tumour invasion, metastasis and poor cancer patient prognosis. Due to VM's important effects on tumour progression, more VM-related strategies are being utilized for anticancer treatment. Here, with regard to the above aspects, we make a review of advanced research on VM in cancer.

- Introduction
- Discovering VM
- Tumour VM formation mechanisms
  –Molecule mechanisms involved in VM formation
  –Other relevant molecule mechanisms
  –CSC, EMT and VM
- VM and cancer therapeutics
- VM and prognosis of human cancer patients
- Conclusion

## Introduction

**figure 1:**
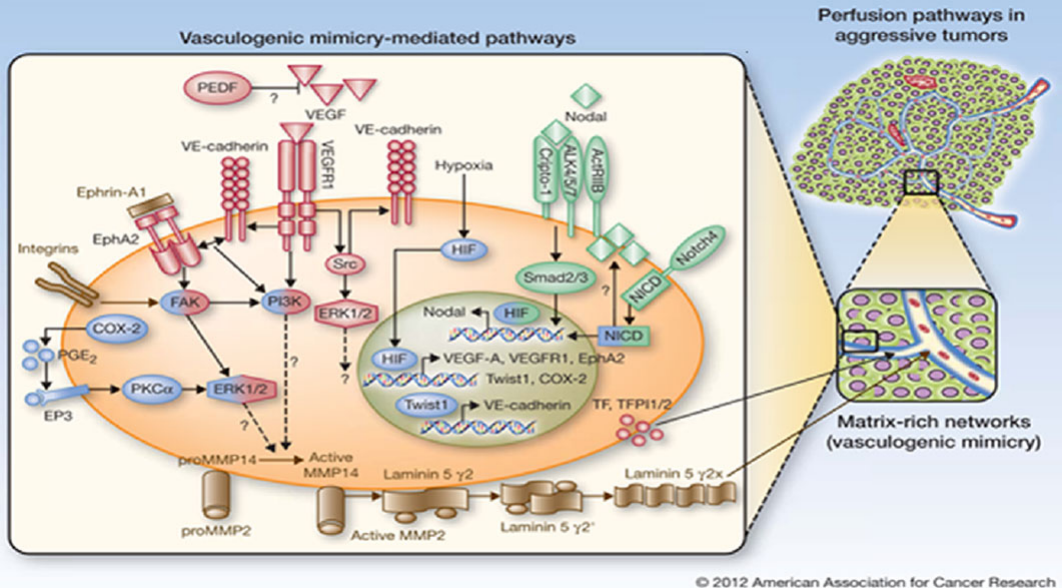
Schematic model of formation mechanisms and signalling pathways in tumour vasculogenic mimicry (VM).

Tumour angiogenesis plays a significant role in tumour growth and metastasis, which was formerly considered the unique choice for tumour blood supply 1–4. Further research has introduced a new channel pattern called ‘vasculogenic mimicry (VM)’, which describes the functional plasticity of aggressive cancer cells in expressing a multipotent, stem cell-like phenotype and forming extracellular matrix (ECM)-rich and patterned vessel-like networks in three-dimensional matrix. Human melanoma cells’ VM is initially characterized as showing that the tumour cells co-expressing endothelial and tumour markers could form vascular channel structures. This provides growing tumours with sufficient blood perfusion and at the same time promotes cancer metastasis and progression 5–7. Besides human melanoma 8,9, VM has been observed in other malignant tumours 10–12, including glioblastoma 13–16, gallbladder cancer 17,18, ovarian cancer 19–21, lung cancer 22, hepatocellular cancer (HCC) 23, breast cancer 24, prostate cancer 14, osteosarcoma 25 and gastric cancer 26,27. More studies aimed to investigate VM's formation mechanisms and signalling pathways (Fig.1) 28, including some factors related to tumour cell migration, invasion and matrix remodelling, such as vascular endothelial-cadherin (VE-cadherin) 29,30, epithelial cell kinase (EphA2) 10,31,32, phosphoinositide 3-kinase (PI3K), matrix metalloproteinase (MMPs), laminin 5 (Ln-5) γ^2^ chain 33–36, hypoxiainducible factor1-α (HIF-α) 37 and focal adhesion kinase (FAK) 38,39. However, the exact mechanisms remain unclear. Additionally, a poor cancer patient clinical outcome was shown to be correlated with VA formation in malignant tumour tissues, and cancer patients with VM tended towards tumour metastasis and had a lower survival rate. VM may be an important target for anticancer therapy, so further studies are needed to improve cancer treatment.

## Discovering VM

Malignant solid tumours need blood supply to promote growth and metastasis. In the past, angiogenesis, one type of neovascularization in embryo development, was regarded as the only form of supporting tumour blood supply. Angiogenesis was first used by British surgeon John Hunter in 1787 to describe blood vessel growth in reindeer antlers. In 1966, Warren first described angiogenesis in melanoma, and afterwards it was investigated in other tumours 40,41. Some studies suggested that angiogenesis was associated with malignant tumour growth and survival 42. Anti-angiogenesis therapies targeting endothelial cells as a potential and promising treatment target have received much attention and investigation. Many anti-angiogenesis drugs have been used to prevent tumour growth and metastasis. However, the drugs’ effects on anticancer progression was restricted and unsatisfactory, which indicated that there may be other blood supply forms in tumour tissues besides angiogenesis. In 1999, Maniotis *et al*. 5 reported a highly patterned vessel-like channel structure in highly aggressive and metastatic human melanoma in which redblood cells were detected. Interestingly, endothelial cells were not identified in these channels by light microscopy, transmission electron microscopy or immunohistochemical detection of endothelial cell markers such as Factor VIII-related antigen, CD34, CD31, Ulex and KDR [Flk-1]. Subsequently, they found that this structure, different from angiogenesis, was comprised of a basement membrane and mainly lined by tumour cells. Melanosome and pre-melanosome were observed in these cells, rather than endothelial cells, showing positive periodic acid-schiff (PAS) and negative CD31 staining using PAS and CD31 staining methods. The vessel-like structures were called ‘VM’. The features of positive PAS and negative CD31 are regarded as the golden standard for tumour cell-lined VM. Generally, VM is divided into two distinctive types: tubular and patterned matrix types 16. VM formation has been found in various highly aggressive tumours in humans, but it was not generated in poorly aggressive melanoma and normal melanocytes under the same culture conditions *in vitro*. Additionally, some researchers also confirmed that VM provides an important perfusion pathway for malignant tumours through sufficient blood and nutrition supply, playing a significant role in tumour growth.

Vasculogenic mimicry is linked with numerous tumour malignancies, including invasion and metastasis 5,43,44. Relevant research verified that there exists a junction in tumour tissues between tumour-lined vascular channels and endothelial-lined blood vessels 45–47. Through this structure, tumour cells lining the inner network channel surface are directly exposed to the blood, significantly increasing transfer opportunities.

Generally, VM characteristics can be summarized as follows: (*i*) positive PAS and negative CD31 straining; (*ii*) the channel is lined by tumour cells rather than endothelial cells; (*iii*) the expression of a multipotent, stem cell-like phenotype; (*iv*) ECM remodelling and (*v*) VM has connection with the tumour microcirculation system, providing blood for tumour growth. Major VM discoveries are illustrated in Table1.

**Table 1:** The major discoveries in the field of VM

Classification	Discovery of VM	References
Structure	Be lined by tumour cells rather than endothelial cells	5
	Remodel the extracellular matrix	28,33,38,39
Histochemical stain	Be positive PAS and negative CD31 straining	5
Epigenetics	Express a multipotent, stem cell-like phenotype	113,114,118,119
Physiological function	Provide blood for tumour growth	5–7
Biological behaviour	Be associated with tumour invasion, metastasis	5,43,44
Clinical application	Be utilized for anticancer therapy	37,133–138
	Be linked with poor prognosis and shorter 5-year survival	22,23,43,139–143

## Tumour VM formation mechanisms

The roles of major adhesion molecules and other factors in VM formation are shown in Table2.

**Table 2:** The roles of major adhesion molecules and other factors in VM

Formation mechanisms	Functions in VM formation	References
MMPs, Ln-5 γ2 chain	The activation of MMPs motivates the cleavage of Ln-5γ^2^ chain into pro-migratory γ^2^ and γ^2x^ fragments which participate in the plasticity of matrix, migration, invasion and formation of VM	28,33,49
PI3K/Akt	PI3K/Akt signal pathway participates in VM formation by regulating the activity of MT1-MMP, MMP-2 and the cleavage of Ln-5γ^2^ chain	35
VE-cadherin, EphA2	VM-cadherin mediates the activities of EphA2, and the phosphorylation of it could activate PIK-3 which promotes VM formation by MMPs and Ln-5 γ2 chain	28,29,31,50
FAK	FAK activates ERK1/2 which mediates MMPs, thus participating in the plasticity of matrix, migration, invasion and VM formation	38,39
TFPI1/2	TFPI-1 is associated with perfusion of VM by its anticoagulant function; TFPI2 through the interaction with MMP-2 was involved in endothelial-cell matrix remodelling and VM formation	58
VEGF, VEGFR1/2	VEGF-A upregulates VE-cadherin, EphA2 and MMPs expressions; VEGFR2 expression contributes to the formation of capillary-like structures (VM)	59–64
Hypoxia, HIF-1α	Hypoxia promotes VM formation by inducing EMT; HIF-1α activates expression of VEGF, and the latter is related to VM formation	20,36,66,67
Gal-3	Gal-3 mediates the expression of VE-cadherin and MMP-2 which have been confirmed to promote VM formation. Silencing of Gal-3 leads to the inhibition of VE-cadherin and IL-8 promoter activities	74
cAMP	The increase of cAMP results in inhibition of VM formation through activation of Epac/Rapl pathway and inhibition of MMP-2 and MT1-MMP expression	
Nodal	Activation of Nodal contributes to VM formation by increasing VE-cad expression. And inhibition of VM formation could be inhibited *via* the activation of Nodal signal mediated by cAMP	77
COX2	COX-2 results in up-regulation of VEGF expression by activating PKC, and PGE-2 expression, thereby promoting VM formation	28,79
CSC, EMT	CSC and EMT are associated with VM formation. And CSC may be implicated in VM formation by EMT induction	113,118,119,125,126,128–132

VM, vasculogenic mimicry; MMPs, matrix metalloproteinase; Ln-5 γ2, laminin 5 (Ln-5) γ^2^ chain; PI3K, phosphoinositide 3-kinase; EphA2, epithelial cell kinase; FAK, focal adhesion kinase; TFPI1/2, tissue factor pathway 1/2; VEGF, vascular endothelial growth factor; VEGFR1/2, vascular endothelial growth factor receptor 1/2; HIF-1α, hypoxiainducible factor1-α; Gal-3, galectin-3; cAMP, cyclic adenosine monophosphate; COX2, Cyclooxygenase; CSC, cancer stem cell; EMT, epithelial-mesenchymal transition.

### Molecule mechanisms involved in VM formation

#### PI3K/Akt, MMPs and Ln-5 γ2 chain

PI3K is a lipid kinase that acts through the phosphorylation process of its substrates, mainly including phosphatidylinositol or its derivatives. The PI3K signal pathway has been shown to be imperative in normal cell processes like proliferation, differentiation, survival, metabolism and motility 48. Akt, also known as protein kinase B (PKB), is a serine/threonine PK that plays an integral role in the PI3K signal pathway. PI3K's products after activation, including PI-3,4-P2 and PI-3,4,5-P3, could combine with Akt's pleckstrin homology (PH) domain. This leads to Akt's translocation from the cytoplasm to the cell membrane and its conformational change, further promoting its activation. It has been confirmed that PI3K/Akt pathway could inhibit cell apoptosis by multiple processes to promote cell survival and tumourigenesis. The main processes included are as follows. Firstly, activation of Akt phosphorylates Bcl2-antagonist of cell death (BAD) on Ser136, a pro-apoptotic Bcl-2 related protein, to effectively block apoptosis 49,50. Secondly, caspase-9 acting as an initiator and an effecter of apoptosis, could be phosphorylated on Ser196 by Akt, further inhibiting apoptosis and promoting cell survival 51,52. Thirdly, Akt contributes to the regulation of cell survival through transcriptional factors such as Forkhead, NF-κB and p53, which are responsible for pro- and anti-apoptotic genes 53–57. Additionally, recent studies have reported that PI3K/Akt signal pathway also participates in VM formation by regulating the activity of membrane type 1 (MT1)-MMP, MMP-2 and the Ln-5γ^2^ chain's cleavage 35.

Membrane type 1-MMP and MMP-2 seem to be crucial to VM formation. MMP overexpression in human ovarian cancers helps form a vascular system lined by tumour cells 36. PI3K/Akt mediates MT1-MMP's function, and the latter could promote pro-MMP's transition into its active form by coactions with the tissue inhibitor of MMP-2 (TIMP-2). Then, MMP-2 activation leads to Ln-5γ^2^ chain cleavage into pro-migratory γ^2^ and γ^2x^ fragments. These fragments are deposited in the ECM and participate in matrix plasticity, migration, invasion and VM formation 28,33. Researchers found that the poorly invasive melanoma cells inoculated in collagen matrix, after pre-treatment with highly invasive melanoma cells, could form tubular network structures along enriched Ln-5γ2 chain tracks deposited in invasive malignant cells. The findings demonstrated that PI3K/Akt, MMPs and Ln-5γ^2^ chain contribute to ECM remodelling and VM formation.

The inhibition of PI3K/Akt as well as related genes by gene knockout or drugs intervention to promote cancer cell apoptosis has been the focus of treatment of tumour, like the application of siRNA, antisense oligonucleotides and small molecule inhibitors. Recently, antiangiogenesis therapies by inhibiting the PI3K/Akt signal pathway and MMPs activation have become another hot research topic in the field of anticancer therapy. Undoubtedly, the combination of anti-apoptosis and anti-VM formation by inhibiting the PI3K/Akt signal pathway may be an important target of treatment, and further researches are needed to find more effective drugs to suppress tumourigenesis.

#### FAK, EphA2 and VE-cadherin

Vascular endothelial-cadherin is one of the transmembrane proteins in the cadherin family and can be specifically expressed in endothelial cells 58. VE-cadherin, an important adhesive protein, could promote homotypic cell interaction and play a significant role in vasculogenic activities 28. Previous studies showed that VE-cadherin was expressed in aggressive melanoma cells but not in non-aggressive melanoma cells. Furthermore, VE-cadherin expression knockdown could inhibit VM formation 29, indicating that VE-cadherin may be associated with VM formation. VM-cadherin's function in VM formation mainly acts by mediating EphA2 activities. EphA2 is a member of the ephrin-receptor family of PTKs and expresses in melanoma cells with a metastatic phenotype, which is crucial to angiogenesis 59,60. Microarray analysis showed that EphA2 and VE-cadherin were overexpressed in human aggressive melanoma cells but not in poorly aggressive melanoma cells 29,31. EphA2 and VE-cadherin are co-localized in sites with cell to cell contact. EphA2 knockdown could make cells lose their VM-forming abilities and lead to EphA2's redistribution in the cell membrane, but it did not affect the positioning of VE-cadherin in cell – cell adhesion 58. The interaction of EphA2 and its membrane-bound ligands will lead to EphA2's phosphorylation, which could activate PIK-3 through the FAK and ERK1/2. Finally, MMP-2 activation and Ln-5γ^2^ cleavage results in VM formation.

Focal adhesion kinase is a cytoplasmic tyrosine kinase linked with focal adhesion 58. Relevant studies showed that FAK could regulate VM migration, invasion and formation in malignant tumours. When FAK is positioned on a membrane, it can activate extracellularly signal-regulated kinase1and2 (ERK1/2). ERK1/2's phosphorylation further mediates MT1- MMP and MMP-2 through the PI3K signal pathway, and is thus involved in the ECM plasticity, migration, invasion and VM formation 38,39.

#### TF, TFPI-1 and TFPI-2

Tissue factor (TF) is a transmembrane protein expressed in many cell types, including endothelial cells, smooth muscle cells, macrophages and solid tumours 61–64, and is related to vascular system development 65,66. TF pathway 1 (TFPI1) and TF pathway 2 (TFPI2) are two coagulation pathway inhibitors, playing an important role in maintaining coagulation and anticoagulation system balances. A recent study demonstrated that TF, TFPI-1 and TFPI-2 were overexpressed in human invasive melanoma cells 67. TF's procoagulant function could be mediated by TFPI, which has been shown to have connections with VM network structure perfusion. Conversely, TF's function cannot be inhabited by TFPI-2, but it is still regarded as an important VM formation factor. TFPI2, through interaction with MMP-2 and the plasmin-dependent way, was involved in endothelial-cell matrix remodelling. MMP-2 activity can be suppressed by inhibiting TFPI-2 expression. Additionally, several studies indicated that endothelial and tumour cell adhesion and migration could be regulated by matrix-related TFPI-2 activity. These findings suggested that TFPI-2 provides an essential mechanism for tumour progression and VM formation.

#### VEGF, VEGFR1/2, hypoxia and HIF-1

VEGF-A is secreted by almost all tumour cells and belongs to the angiogenic growth factor family associated with tumour angiogenesis. Binding VEGF-A to its ligand results in the dedifferentiation of endothelial cells into its precursor, stimulating vascular channel proliferation and formation in tumours, especially in avascular regions. Findings showed that, in melanoma, VM and angiogenesis were mediated by VEGF-A 68. Relavant research demonstrated that, in ovarian cancer, expressions of VE-cadherin, EphA2 and MMPs could be up-regulated by VEGF-A, contributing to matrix plasticity and VM formation 69. Silencing VEGF using siRNA could induce cell apoptosis and suppress cell proliferation and VM formation in osteosarcoma 70. Brantley-Sieders 71 observed that VEGF expression and angionenesis induced by VEGF were decreased through repressing EphA2 activity in breast cancer. The result also was obtained by another study on pancreatic islet cells.

The two tyrosine kinases receptors, VEGFR1 and VEGFR2, bind to VEGF and possess different characteristics. VEGFR1 has a higher VEGF binding ability but lower kinase activity compared to VEGFR2. VEGFR2 is linked with tumour angiogenesis 72,73. In the PI3K/Akt signal pathway, VEGFR1 activation was considered to participate in endothelial cell differentiation and angiogenesis. Melanoma cells failed to form capillary-like structures when VEGFR1 expression was inhibited using siRNA. This result further identifies the requirement of VEGFR1 for VM. However, inhibiting VEGFR2 expression by a specific inhibitor PTKI could not affect capillary-like structure formation, indicating that VM formation was mainly influenced by VEGFR1 activity rather than VEGFR2.

Growing tumours lack blood supply in a normal state. Hypoxia can stimulate tumour vessel structure formations 74 including angiogenesis and VM, which contributes to tumour metastasis. After hypoxia develops, HIF-1α and HIF-2α are generated by tumour cell activation. Then, HIF-1α activates VEGF expression by binding to the VEGF gene enhancer sequence, thereby resulting in VM formation 37,75,76. Du *et al*. 20 showed that hypoxia promoted VM formation by inducing epithelial-mesenchymal transition (EMT) in ovarian cancer. In highly invasive gallbladder cancer cells, hypoxia could reportedly induce VM formation and increase HIF-1α expression. HIF-1α knockdown by siRNA significantly inhabited HIF-1α expression and VM channels under normoxia or hypoxic conditions 17. Furthermore, in Ewing sarcoma tumours, HIF-1α activation was also shown to participate in VM formation. A recent study demonstrated that HIF-1α localization is not along the CD-31 zone of blood vessels but the VM networks 77, which was verified by hypoxia marker pimonidazole staining. Furthermore, severe hypoxia was found in tumour VM zones. These results suggested the relation between hypoxia and VM formation.

#### Galectin 3

Galectin-3 (Gal-3) is a β-galactosyl-binding lectin involved in biological functions including cell adhesion, cell migration, cell apoptosis and angiogenesis 78,79. Gal-3 is composed of three distinct domains: a glycine and proline-riched repeated collagen-like sequence domain, an NH2-terminal domain and a COOH-terminal carbohydrate recognition domain 80–82. Gal-3 is widely expressed in various highly invasive tumours but not found in normal cells and most benign tumours. During benign melanoma's progression to metastatic melanoma, Gal-3 was accumulated in tumour cell cytoplasm and in turn stimulated tumour cells to transform into higher invasive types and resulted in vessel formation and distant metastasis. Mourad-Zeidan AA 83 showed that Gal-3 silencing using shRNA made melanoma cells lose their invasiveness abilities and channel structure formation on collagen *in vitro*. Gal-3 mediated the expression of many genes, such as VE-cadherin, interleukin-8 (IL-8) and MMP-2, which are confirmed to contribute to VM formation. Silencing Gal-3 expression inhibited VE-cadherin and IL-8 promoter activities, mainly owing to increased recruitment of early growth response-1 (EGR-1), a transcription factor participating in maintaining differentiation processes in normal tissues. EGR-1 overexpression repressed VE-cadherin, IL-8 and their promoter activities, while Gal-3 expression blocked EGR-1 binding to gene promoter and induced VM formation in tumour tissues.

#### Cyclic adenosine monophosphate and nodal

Cyclic adenosine monophosphate (cAMP), a second messenger regulating cell growth and differentiation, is generated by activated adenylyl cyclase acting on ATP. Several studies have proven that in aggressive melanoma, cAMP increase resulted in VM formation inhibition through multiple signalling pathways. First, cAMP stimulates PKA-independent activation of Epac/Rapl pathway to restrain vessel-like channel structure formation. Additionally, cAMP helps inhibit MMP-2 and MT1-MMP expression by suppressing ERK1/2 to block VM formation. A recent study found that forskolin resulted in increased cAMP that strongly restrained VM formation by activation of Epac/Rap1, and inhibition of ERK1/2 and PI3K/Akt functional activation 84.

Nodal belongs to one transformation growth factor β (TGF-β) superfamily and plays an important role in maintaining tumourigenicity and melanoma progression. Nodal and Notch receptor were implicated in stem cell-associated plasticity development and up-regulation of Nodal expression, which was found in invasive melanoma cells 85. Topczewska *et al*. 86 demonstrated that activating Nodal contributed to VM formation by increasing VE-cadherin expression. Inhibiting Nodal expression prevented VM formation and melanoma cell invasiveness by decreasing keratin and VE-cadherin. Additionally, cAMP, through VE-cadherin redistribution, was able to enhance endothelial barrier properties and promote cell adhesion. VM formation could be inhibited by activating the Nodal signal mediated by cAMP 84. Therefore, cAMP and Nodal are likely to participate in VM formation, and more relevant studies are crucial to find an effective anticancer therapy target.

#### COX2

Cyclooxygenase (COX), a necessary enzyme in prostaglandins synthesis, consists of the isoenzymes COX-1 and COX-2. A house keeping enzyme, it mainly existed in blood vessels, stomach and other tissues. It is widely regarded as participating in the body's normal physiological processes and protection functions, such as maintaining gastrointestinal mucosa integrity and platelet function adjustment. The latter is an inducible type mainly produced by inducting various pathophysiological stimuli including chemical, physical and biological factors, thus participating in inflammatory reactions by promoting prostaglandins synthesis. As a rate-limiting enzyme of prostaglandin E2 (PGE2), COX2 shows no expression or low expression in normal tissue cells, but it is highly expressed in a variety of epithelial tumours due to environment induction. Basu *et al*. 87 found that human invasive breast cancer cells with high COX-2 expression were observed to form VM in three-dimensional culture, while breast cancer cells with lower COX2 expression failed to form vessel-like channel structures. COX-2 knockdown using siRNA in highly invasive tumours markedly restrained vessel structure formation, indicating that COX-2 may contribute to VM formation regulation. Additionally, COX-2 resulted in the up-regulation of VEGF expression by activating PKC in various tumour cells, thereby promoting VM formation 88. COX-2 can also increase PGE-2 expression, and both are overexpressed in invasive tumours and associated with tumour proliferation, apoptosis, invasion and angiogenesis by regulating the prostanoid receptor (EP1-4) family 28. Binding PGE2 with its ligand EP3 could activate ERK1/2 by PKC to mediate VM formation. Therefore, COX2 may play an important role in VM formation. Its specific mechanisms need further research.

### Other relevant molecule mechanisms

#### Inhibitors of DNA binding 2

Inhibitors of DNA binding 2 (Id2) is a member of the helix-loop-helix (HLH) protein family that participates in many cellular processes, including cell proliferation, differentiation, cell cycle regulation and tumourigenesis 89–91. In highly aggressive uveal melanoma cells, Su 92 reported that Id2 knockdown using RNA interference could abolish VM formation by down-regulating VE-cadherin expression. It also affects cellular stability, indicating that Id2 may be vital to vessel-like structure formation.

#### Migration-inducing protein 7

Migration-inducing protein 7 (Mig-7), a cysteine-rich protein, plays an important role in tumour migration and circulation. There is evidence showing that Mig-7 is overexpressed in highly aggressive melanoma cells with VM but not in poorly aggressive melanoma without VM 93. Mig-7 overexpression was capable of increasing Ln-5γ^2^ chain domain III fragments, thus promoting tumour cell migration, metastasis and VM formation.

#### Caspase-3

Caspase-3 is implicated in cell apoptosis 94,95, proliferation 96–98, migration 99–101 and differentiation 102–104 in normal tissues and tumours. In melanoma metastases, active caspase-3 expression was observed 105. Liu *et al*. 106 revealed that downregulating caspase-3 by siRNA helped inhibit tumour migration, invasion and VM, suggesting that caspase-3 is related to tube-like structure formation. Additionally, it has been pointed out that caspase-3 mutations are not uncommon in malignant cancers. Chen *et al*. 107 showed that genetic variation in caspase-3 is associated with an increased risk of squamous cell carcinoma of the head and neck (SCCHN). Most of the caspases mutations detected in human cancers showed attenuate apoptosis compared with the wild-type caspases, indicating a significant role of the inhibition of apoptosis by caspases mutations in tumourigenesis 108. But it seems that there are no correlative studies on the relativity between caspases mutations and tumourigenesis induced by VM. Therefore, considerable studies about caspases and VM are important, and contribute to finding an effective gene therapy or targeting therapy for tumours.

#### Endothelin-1

Endothelin (ET)-1 is considered a practical tumour marker, the secretion of which is increased in a variety of pathological conditions and cancer patients. In primary and metastatic melanoma cell lines, the activation of a G-protein coupled receptor by ET-1 could markedly result in increased VEGFR-3, VEGF-C and VEGF-D expression. Binding ET-1 to VEGF-C was able to enhance the phosphorylation of VEGFR-3, MAPK and ATK, thereby significantly promoting tumour cell invasion and VM formation 109. Furthermore, the ETBR antagonist repressed VEGF-C/VEGFR-3 axis activation and prevented tumour growth and VM formation, suggesting that ET-1/ETBR might provide an essential therapeutic target for melanoma treatment by VEGFR-3.

#### Bone morphogenetic protein 4

Bone morphogenetic protein 4 (BMP4) is another tumour migration and invasion regulator. Reducing BMP4 activity in melanoma cells reduced VE-cadherin and EphA2 expression and failed to promote tubular structure formation 110.

#### Human chorionic gonadotropin

Human chorionic gonadotropin (hCG) is reportedly capable of inducing VM formation in ovarian cancer cell line OVCAR-3. hCG expression is higher under hypoxia compared to normoxia. Inhibiting HIF-1α expression using siRNA resulted in significantly decreased hCG expression in OVCAR-3. Treating OVCAR-3 with 5000 mU/ml hCG led to the induction of vessel-like networks lined by tumour cells and markedly enhanced vascular marker expression even under normoxia 21.

### CSC, EMT and VM

Tumours contain a small portion of cells capable of self-renewal and multiple potential differentiation called cancer stem cells (CSCs). Lapidot *et al*. 111 firstly demonstrated CSC, and they were subsequently observed in several solid tumours, including breast cancer 112, glioblastoma 113, colon cancer 114, melanoma 115,116, ovarian cancer 117,118, prostate cancer 119 and pancreatic cancer 120. More evidence shows that CSCs are capable of differentiating towards tumour and endothelial lineages 121,122, possessing both phenotypes. Additionally, CSCs are associated with tumour invasion and metastasis, and many CSC markers were involved in these processes, such as ALDH1 123, CD44^+^ 124 and FRMD4A 125. Hermann *et al*. 120 also showed that CSCs phenotypes of CD133/CXCR4 in pancreatic cancer are related to tumour metastasis and tumourigenesis, and CSCs are thus classified into two types: invasive CSCs crucial in tumour invasion and metastasis, and stationary CSCs associated with tumourigenesis.

Recently mounting studies implicated CSCs in VM formation. Mirshahi *et al*. 126 found that adherent bone marrow stromal cells derived from CD133^+^/CD34^+^ stem cells from acute leukaemia patients were able to secret more insulin growth factor-1 and stromal cell-derived factor-1 (SDF-1) alpha, and could result in the formation of capillary-like structures (‘VM’) on Matrigel. Furthermore, there is evidence showing that in oral SCC, TRA-1-60^+^/beta6^+^ stem cells were capable of producing vascular-like structures *in vivo*. Additionally, in melanoma, it was shown that some phenotypes generally expressed in epithelial or endothelial cells were observed to express in VM-engaging tumour cells 127. Ricci-Vitiani *et al*. 121 found that in glioblastoma, the vessels in tumour xenografts generated by orthotopic or subcutaneous injection of glioblastoma stem-like cells in immunocompromised mice were made up of human endothelial cells, which indicated CSCs’ differentiation potential along endothelial lineage and their involvement in VM formation.

Epithelial-mesenchymal transition is a dedifferentiation process that plays an integral role in tumour progression. By epithelial cells transitioning into mesenchymal cells, EMT acquired mesenchymal features and lost epithelial phenotypes, mainly including epithelial marker downregulation, mesenchymal marker upregulation and cell polarity loss. EMT is reportedly involved in tumour invasion and metastasis. Cadherin switching, an important EMT marker associated with the EMT differentiation process, contains E-cadherin expression loss and N-cadherin gains. Recent studies show that transcription factors related to EMT are upregulated in VM-forming tumour cells. As a main EMT-mediated process regulator, Twist reportedly promotes breast cancer metastasis into distant regions 128. Similarly, in HCC, Zhao *et al*. 129 found that Twist1 could down-regulate E-cadherin expression and contribute to MMP activation, particularly in MMP2 and MMP9, thus inducing HCC invasion. Interestingly, Twist2 had no effect in HCC invasion and metastasis, and specific mechanisms need further research. Snail and Slug are two other transcription factors able to inhabit E-cadherin transcription and stimulate tumour cell migration by binding to E-boxes present in human E-cadherin promoter 130–132. A recent study demonstrated that EMT was implicated in VM formation. It showed that the poorly differentiated cell line SK-Hep-1 with mesenchymal features (high invasiveness and expressing Vimentin, with no E-cadherin) was capable of forming VM *in vitro*. The well-differentiated cell line HepG2 failed to form VM, and no correlation was found between intrinsic VM ability and stemness gene expression 133. In colorectal carcinoma, Liu *et al*. 134 showed that ZEB1 expression was upregulated in VM-positive samples compared with VM-negative samples, while it occurred concurrently with EMT traits. Additionally, ZEB1 knockdown in tumour cells abolished VM formation, led to epithelial phenotype restoration, and evidently repressed tumour migration and invasion. Moreover, ZEB1 down-expression resulted in decreased VE-cadherin expression and Flk-1, which went against VM formation. This means EMT contributes to VM formation and maybe provides a therapeutic target for anti-angiogenesis therapy.

With increasing studies on CSC's origin, more evidence indicated that differentiated tumour cell stemness can be acquired by EMT induction, which may become a new CSC origin mechanism. Xia *et al*. 135 reported that miR-200a could regulate EMT by targeting ZEB2 and stem-like transition by β-catenin signalling in nasopharyngeal carcinoma cells. Stable miR-200a knockdown stimulated EMT phenotypes and led to stem cell characteristics, including sphere formation capacity, CD133 (+) side population, stem cell marker expression, and *in vivo* tumourigenicity in nude mice. Stable miR-200a overexpression had the opposite result. Fang *et al*. 136 showed that Twist2 overexpressed in breast carcinoma cells promoted stem cell marker expression, increased stem-like cells’ self-renewal abilities, and contributed to tumour progression. It was also found that epithelial breast cancer cells undergoing EMT acquired a CD24(-/lo)CD44(+) phenotype consistent with breast CSCs. They also acquired breast CSC features, including epithelial tumour reestablishing capacity, potent tumourigenicity and increased resistance to drugs and radiation 137. Furthermore, in mammary tissue, Mani *et al*. 138 observed that mesenchymal traits and stem-cell marker expressions could be obtained by EMT induction in immortalized human mammary epithelial cells (HMLEs). Moreover, stem-like cells isolated from HMLE cultures or mammary carcinomas could express EMT markers. The relationship between EMT and CSC has also been found in HNSCC 139 and colorectal 140. Additionally, the hypoxia microenvironment is considered an important factor in regulating VM formation by maintaining stemness and EMT induction. These findings widely suggested that CSC may be involved in VM formation by EMT induction.

## VM and cancer therapeutics

Anti-angiogenic treatment is widely accepted as an effective anticancer therapy. Common anti-angiogenic drugs like angiostatin and endostatin play a role mainly by reducing endothelial cell proliferation or inducing endothelial cell apoptosis, but they have little effect on vessel-like structures lined by tumour cells. Furthermore, when blood vessel density is reduced due to anti-angiogenic therapy, it may lead to hypoxia. Subsequently, oxygen and nutrient deficiency as a compensatory stimulus will contribute to VM formation and indirectly promote tumour progression. Moreover, several of these drugs have a variety of side effects, thus limiting their usefulness in treatment. Therefore, further study is needed to find safe and effective therapies against the invasion and metastasis of highly aggressive tumours.

Recently, mounting studies focus on a new anticancer treatment that inhibits VM formation and is involved in various mechanisms, including antisense oligonucleotides to the Ln-5 γ2 chain, antibodies to MMP-2 or MT1-MMP, VE-cadherin downregulation, and inhibiting other VM-associated genes. Zhang *et al*. 141 showed that thalidomide through inhibiting VEGF, MMP-2 and MMP-9 expression suppressed VM channel and mosaic vessel formations in melanoma. Thalidomide was used in the last century to treat pregnancy reactions but was stopped due to its severe teratogenic effects on the foetus. Perhaps this effect on embryonic cells made thalidomide possess the ability to inhibit vessel structure formation. Besides thalidomide, doxycycline was also reported to contribute to the inhibition of engrafted melanoma progression by decreasing VM formation and MMP-2 and MMP-9 expression. In murine osteosarcoma LM8 cells, Fu 142 demonstrated that zoledronic acid (ZA) could restrain VM development by damaging RohA membrane localization in LM8 cells, resulting in cell ultrastructure changes and stimulating cell apoptosis. Previous data indicated that tetracycline COL-3, after chemical modification, was able to inhabit VM-associated gene expressions in aggressive tumour cells, thus repressing VM formation 143. Celecoxib may restrain vessel-like structure formation by inhabiting COX-2 in human breast cancer. Increased exogenous PGE2 helped abolish vessel-like structures 144. Therefore, it was hypothesized that celecobix may have an effect on vascular structures by PGE2. Additionally, Su *et al*. 37 observed that rapamycin, a HIF-1α inhibitor, was capable of blocking VM formation and phenotype transformation by suppressing VEGF, VE-cadherin, EphA2 and MMP-2 expressions. Itzhaki *et al*. 145 reported that nicotinamide, the amide form of vitamin B3 (niacin), partially repressed VM formation by VE-cadherin downregulation and destroyed those already formed in a dose-dependent manner. Moreover, VM inhibition abilities even lasted for 1 month after complete nicotinamide withdrawal, indicating that nicotinamide targeting VM may become an effective therapy against tumour progression. Besides western medicines, traditional Chinese medicines were utilized to analyse the effectiveness for VM formation and tumour inhibition. In a murine choroidal melanoma model, Yadav and Aggarwal 146 observed that curcumin could inhibit VM formation and tumour growth by downregulating the EphA2/PI3K/MMP pathway. With further studies and a large number of clinical trials, VM inhibitors combined with anti-angiogenic therapies appear to be a promising therapeutic target in anti-tumour therapy.

## VM and prognosis of human cancer patients

Vasculogenic mimicry provides adequate blood supply for various malignant tumours to promote tumour invasion and metastasis. Subsequent studies showed that VM was significantly linked with poor prognosis for patients with aggressive tumours 22,23,43,147. Poor 5-year survival was observed in some VM-forming aggressive cancers, including melanoma, colorectal cancer, lung cancer, sarcomas and hepatic cancer. Cao *et al*. 148 reported that the relative 5-year survival risk of VM-positive patients was significantly higher than with VM-negative cases. Higher VM rates and worse 5-year survival rates were observed in melanoma patients with metastasis compared to patients with primary melanoma, indicating that VM was able to promote tumour metastasis and poor prognosis in malignant cancer patients. In 168 cases of laryngeal squamous cell carcinoma (LSCC), Lin *et al*. 149 demonstrated that VM formation in LSCC cells enhanced tumour invasion and metastasis potential. Additionally, a VM positive rate was strongly related to tumour stage, grade and metastasis, and miR-200a expression in VM-positive ovarian cancer showed low levels. Furthermore, the overall survival (OS) of patients with low miR-200a expression and/or positive VM formation was significantly shortened by evaluating Kaplan–Meier curves 150. The finding suggested that VM and miR-200a might play a vital role in ovarian cancer's progression and prognosis. Recently, Wang *et al*. 151 revealed that the median survival rate of VM-forming patients is markedly shorter than in patients without VM, so VM may be a prognostic factor for post-operative survival in patients with glioblastoma. However, several studies demonstrated that VM had no significantly statistical association with tumour prognosis, although shorter survival was observed in VM-positive patients 43,127,152,153. Therefore, VM's influence on cancer patients’ OS remains controversial. VM is widely considered, though, to provide predictive signals for cancer patient prognosis. Further study is needed to analyse the precise relationship between VM and human cancer patient prognosis, contributing to better individual therapy selection for cancer patients by predicting tumour progression early.

## Conclusion

As a brand-new tumour vascular pattern, VM describes the functional ability of aggressive cancer cells to express a multipotent, stem cell-like phenotype and form ECM-rich and patterned vasculogenic-like networks to provide adequate blood supply for tumour growth in a three-dimensional matrix. VM with positive PAS and negative CD31 is lined by tumour cells and independent of epithelial cells. As a unique perfusion way, VM has been observed in a variety of aggressive tumours. Many molecule mechanisms, especially VE-cadherin, EphA2, PI3K, MMPs, VEGFR1 and HIF-1a, are involved in tumour migration, invasion, and VM formation. However, their specific roles in VM still remain unclear. Furthermore, CSC and EMT are also shown to participate in VM formation. Also, CSC may be implicated in VM formation by EMT induction. There is increasing evidence showing that VM and VM density levels are linked with poor prognosis and shorter 5-year survival in cancer patients. Ordinarily, VM-forming aggressive tumours have the increased potential to metastasize to distant organs, therefore, predicting cancer progression in advance may be extremely important. Currently, more VM-related strategies are utilized for anticancer treatment by preventing VM formation, and many drugs shows an effective outcome in inhibiting it. More therapies targeting VM need to be attempted in experimental and clinical research. VM inhibitors combining with anti-angiogenic therapies may be a promising therapeutic target in anti-tumour therapy.
